# The impact of parental dental anxiety and oral health literacy on child oral health and dental-visit patterns: a cross-sectional study

**DOI:** 10.1186/s12903-024-04536-8

**Published:** 2024-07-27

**Authors:** Ravi Kumar Gudipaneni, Khalid Maziad D. Alzabni, Faisal Fraih A. Alrashedi, Dimah Hamoud J. Alruwaili, Farah Awad Albalawi, Asrar Helal Alanazi, Buthainah Saleh Alshamri, Saud Hamdan Almaeen, Nithin Manchery, Omar A. Bawazir

**Affiliations:** 1 Pediatric dentistry, Department of Preventive Dentistry, College of Dentistry, Jouf University, Sakaka, Al Jouf, Saudi Arabia; 2 Department of Preventive Dentistry, College of Dentistry, Jouf University, Al Jouf, Saudi Arabia; 3 School of Dentistry, The University of Queensland Herston, Queensland, 4006 Australia; 4 Department of Pediatric Dentistry and Orthodontics, College of Dentistry, King Saud University, Riyadh, Saudi Arabia

**Keywords:** Dental anxiety, Self-reported oral health, Oral-health literacy, Pattern of child dental visits, Socio behavioural characteristics, Dental caries

## Abstract

**Background:**

Identifying the risk indicators of parental dental anxiety (PDA) and oral health literacy (OHL) can help oral healthcare professionals recognise challenges in this field. Armed with the appropriate information, they can effectively engage with parents to build trust and promote early and regular child dental visits.

**Objectives:**

This study aimed to investigate the association between PDA and OHL with the sociobehavioural characteristics of families, self-reported child oral health (presence of ≥ 1 untreated decayed teeth) and the dental visit patterns amongst children living in Al Jouf Province, Kingdom of Saudi Arabia.

**Subjects and methods:**

A total of 430 parents with children aged ≥ 14 years were invited using a systematic random sampling method. PDA was assessed using the Dental Anxiety Scale-Revised (DAS-R) scale, and parents’ OHL was measured using the Rapid Estimate of Adult Literacy in Dentistry-30 (REALD-30). The relationships amongst participant characteristics, PDA and OHL were evaluated through the Chi-square and ANOVA. Additionally, binary regression analysis was conducted to identify predictor variables associated with PDA and OHL. A P value of < 0.05 was considered statistically significant.

**Results:**

Children with ≥ 1 untreated decayed tooth were 2.5 times more likely to have PDA (95% confidence interval [CI] = 1.37, 4.37). Children who visited the dentist in < 6 months had 93% lower likelihood to have PDA (adjusted odds ratio (AOR) = 0.07; 95% CI = 0.03, 0.18). Parents aged 20–25 years were 81% less likely to have OHL than those above 30 years (AOR = 0.19; *P* = 0.038). Similarly, parents with medium family income were 52% less likely to have OHL than the high-income group (AOR = 0.48; *P* = 0.013). Finally, parents of children who visited the dentist within < 6 months were 34 times more likely to have OHL than those whose children visited the dentist > 12 months ago (AOR = 34.94; *P* < 0.001).

**Conclusion:**

PDA and OHL were significantly affected by parental age, family income, the presence of ≥ 1 untreated decaying tooth and the child dental visit patterns. During a child’s first dental visit, paediatric dentists should always assess the PDA, OHL and sociobehavioural characteristics of a family by using appropriate scales and semistructured interviews.

## Introduction

Oral health is often neglected and given a lower priority than other global health issues [1]. In Saudi children, dental caries is severe and highly prevalent, affecting 80% of primary teeth and 70% of permanent teeth [2]. Current estimates suggest that children in the Kingdom of Saudi Arabia (KSA) have not yet achieved the World Health Organization’s target goals [2]. In particular, Al Jouf Province in KSA reports an alarmingly high prevalence rate of early childhood caries, with 94.2% of preschool children having one or more decayed teeth and 56.5% with pulp-involved teeth [3].

Dental anxiety (DA) is a common reason for adults missing dental check-ups [4], with 17–25% of parents in KSA experiencing high DA, thus potentially acting as a barrier to dental-care utilisation [4, 5]. In developed countries, the prevalence of DA in the general population ranges from 4 to 20% [6, 7]. Parents who have DA are less likely to take their children to the dentist [8]. As a result, parental DA (PDA) can influence children’s DA [9]. Studies have shown that approximately 10–43% of children experience DA, thus presenting a significant challenge in paediatric dentistry ( [10]. DA is also more widespread amongst parents with underprivileged backgrounds and lower levels of education than their more educated and affluent counterparts [8].

The oral health literacy (OHL) of caregivers also has a significant influence on the oral health outcomes in young children [11]. Caregivers from low-income backgrounds are more likely to have low OHL, high DA and infrequent use of oral healthcare services [12]. The main obstacles to accessing oral healthcare services are financial constraints, geographic location and low levels of OHL [12]. A nationwide cross-sectional study conducted in the KSA has identified risk indicators for healthcare utilisation, including low health literacy, older age, lower income and educational level [13]. Poor health literacy increases the risk of dental diseases, leading to higher healthcare costs [14]. Furthermore, low OHL amongst caregivers results in poor oral health behaviours in young children, including night-time bottle feeding and irregular brushing habits [15]. Income inequality has also been associated with poor oral health in various national studies conducted in the United States, Japan and Brazil [6].

In the KSA, a significant prevalence of extreme PDA has been reported (21.6%) [5]. However, evidence is limited with regard the factors influencing parental or caregiver DA and OHL and how they relate to their child’s dental health and dental visit patterns amongst Saudi children. Thus, the present study hypothesises that PDA and OHL are significantly associated with child oral health, sociobehavioural characteristics and the dental visit patterns in children. Previous research has suggested that OHL and DA are related. A number of interrelated factors, such as inadequate OHL and DA, can reportedly explain the poor oral health of socioeconomically disadvantaged patients [16, 17]. As such, public health programs should place a high priority on effective communication techniques and anxiety management to improve oral health in this population. In this regard, creating strategies that consider parents’ OHL levels and effectively reduce dental fear is imperative.

Furthermore, understanding these predictors of PDA and OHL associated with child oral health is crucial for policymakers to comprehend the impact of sociobehavioural characteristics and to effectively communicate with parents to help improve their children’s oral health. Accordingly, the present study aimed to investigate the association between PDA and OHL with specific sociobehavioural characteristics (e.g. parent’s age and gender, maternal education and family income), child dental visit patterns and self-reported child oral health (including the presence of ≥ 1 untreated decayed teeth) amongst children residing in Al Jouf Province, KSA.

## Subjects and methods

### Study setting and sample population

This cross-sectional study was conducted in Al Jouf Province, located in the northern border region of the KSA. The participants comprised Saudi parents or caregivers with children aged 12–14 years old or younger. The study was conducted between February and August 2022. Ethical approval (02-05-43) was received from the Local Committee of Bioethics, Jouf University, KSA. The study adhered to the STROBE guidelines and Helsinki Declaration. Furthermore, written informed consent was obtained from all participants.

### Conceptual research framework

**Fig. 1:**
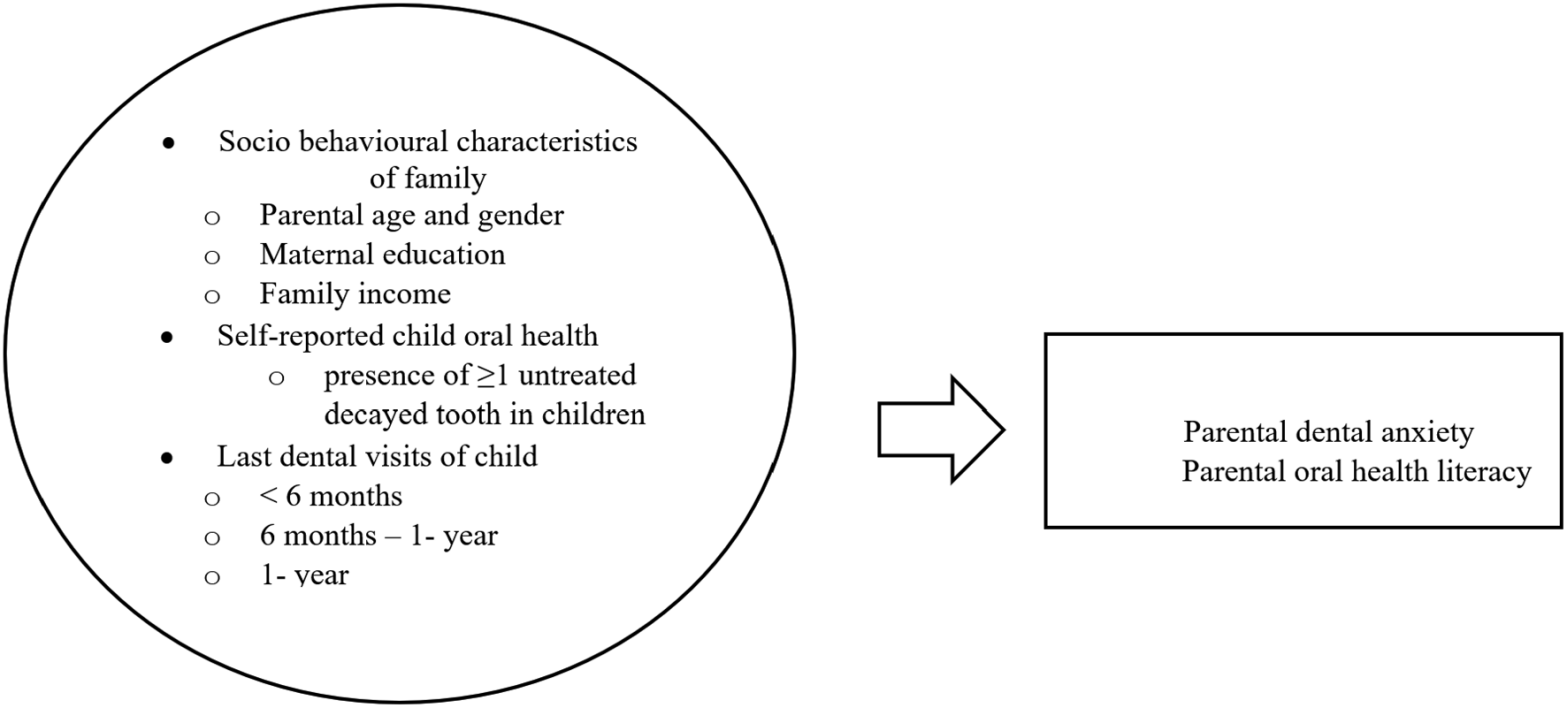
Conceptual research framework

This study is based on the hypothesis that family sociobehavioural variables (e.g. parents’ age and gender, maternal education and family income), child dental visit patterns and self-reported child oral health status (presence of ≥ 1 untreated decayed teeth) are linked to PDA and OHL concerning child oral health (Fig. 1).

### Sample size calculation

The minimum sample size for this study was calculated using specific parameters, including the reported prevalence of parental anxiety at 21.6% amongst the Saudi population [5] with a 5% precision, a statistical significance level of 5% (two-tailed) and a 95% CI. Based on these criteria, the minimum required sample size was determined to be 261 participants. Considering a predicted dropout rate of 10%, the final sample size was set at 290 participants. However, for the study findings to be representative of the Al Jouf population, a total of 430 Saudi parents were invited to participate in the study.

### Sampling technique

Parents attending the university dental clinics of the College of Dentistry, Jouf University, KSA, were invited using a systematic random sampling method. A random starting point was identified, and a fixed sampling interval was determined by dividing the estimated number of children attending the dental clinics each day. The fixed sample interval was set at three, so the parents of every third child were invited to participate in the study. This approach ensured that the study population was randomised and representative of the population living in Al Jouf Province. The inclusion criteria of this study were as follows: parents/caregivers accompanying their children under the age of 14 years and those who provided written informed consent prior to their participation.

### Data collection

Parents or guardians were provided with an Arabic version of a closed-ended questionnaire for data collection. The questionnaire items were initially developed in English and later translated into Arabic by a bilingual translator who was a native Arabic speaker. Subsequently, the translated version was reviewed by another independent bilingual translator who was also a native Arabic speaker. To assess the reliability of each questionnaire item, a subset of 10% (43) of the total sample was randomly selected, the items of which were reevaluated a week later. The internal consistency of all questionnaire items was found to be high, with a Cronbach’s alpha value of 0.87.

The questionnaire comprised three sections. Section 1 covered the sociodemographic and behavioural characteristics of the family, including parents’ age and gender, maternal education, family income, self-reported child oral health (presence of ≥ 1 untreated decayed teeth) and child dental visit patterns. Family income was divided into three categories: low (less than 3,000 SAR per month), middle (3,000–10,000 SAR per month) and high (more than 10,000 SAR per month) socioeconomic status (SES) [18].

Section 2 assessed PDA using the Dental Anxiety Scale-Revised (DAS-R) scale [19]. The DAS-R scale contained four situations related to dental visits, and the participants rated their level of DA on a 5-point scale (1 = relaxed, 5 = high anxiety) for each situation. The total DAS-R score was determined by summing the responses for all four items. The DAS-R is a valid and reliable tool for the rapid evaluation of DA [20].

Section 3 involved assessing parental OHL by using the Rapid Estimate of Adult Literacy in Dentistry-30 (REALD-30) scale [21]. This tool required participants to read aloud 30 dental words that are arranged in ascending order of difficulty based on word length, syllables and sound combination. Each correctly pronounced word earned one point, whereas incorrectly pronounced words were given a score of zero. The sum of scores generated a final score ranging from 0 (lowest literacy level) to 30 (highest literacy level). The REALD-30 tool is advantageous because it takes only 2–3 min to administer and has been correlated with educational backgrounds, oral health awareness, behaviours, perceived need for oral care, dental visits and access to dental care [15]. It is also considered a reliable and accurate method to assess OHL [12].

### Statistical analysis

Statistical analyses were performed, including descriptive and inferential methods. Descriptive statistics, such as mean, standard deviation, frequencies and percentages, were used to summarise the data. For inferential analysis, one-way ANOVA was used to assess the mean differences in OHL based on participant characteristics. Chi-square exact test was also used to determine the association between participant characteristics and PDA, as measured by DAS-R (categorised as mild, moderate, high and severe). Next, multiple logistic regression analysis was conducted to identify significant predictor variables associated with PDA and OHL. Initially, simple logistic regression analysis was used to obtain the crude odds ratio (COR) of potential predictors. Those with P-values less than 0.25 were considered significant factors and were included in the multiple logistic regression. The multiple logistic regression used forward LR and backward LR methods to obtain the adjusted odds ratio (AOR) of the predictors. The final model was generated using the enter method. All statistical analyses were performed with Statistical Product and Service Solution version 26 (IBM, Armonk, NY, USA).

## Results

Table 1 presents the descriptive characteristics of the study participants. A significant proportion of parents (49.1%, *n* = 211) were male and above 30 years old. More than half of the participants (56.7%, *n* = 244) came from middle-income families, and a majority (72.6%, *n* = 312) had maternal education at the graduate level. Furthermore, the majority of parents reported that their child had at least ≥ 1 untreated decayed tooth (83.3%, *n* = 358), and approximately half of the study participants (52.5%, *n* = 227) mentioned that their child’s most recent dental appointment was between 6 and 12 months ago.

**Table 1:** Characteristics of the study participants

Variables	*n* (%)	
Relationship to Child		
Mother	144 (33.5)	
Father	211 (49.1)	
Caregiver/guardian	75 (17.4)	
Age of parent (years)		
20–25	41 (9.5)	
> 25–30	178 (41.4)	
> 30	211 (49.1)	
Family income		
Low	9 (2.1)	
Medium	244 (56.7)	
High	177 (41.2)	
Maternal education		
Primary	7 (1.6)	
High school	111 (25.8)	
Graduate	312 (72.6)	
Presence of ≥ 1 untreated decayed teeth		
Yes	358 (83.3)	
No	72 (16.7)	
Last child dental appointment		
< 6 months	52 (12.1)	
6 months – 1 year	227 (52.8)	
> 1 year	151 (35.1)	

Table 2 presents the significant associations between the independent variables and PDA measured by DAS-R. Parents aged 25–30 years and those from low-income families had a higher prevalence of severe DA (23% and 66.7%, respectively). In contrast, the high-income group showed a higher prevalence of mild DA at 46.9%. Parents with primary and secondary education levels had a higher frequency of moderate DA at 42.9% and 37.8%, respectively, in comparison to the group with graduate level of education, in which parents exhibited a higher prevalence of mild anxiety at 39.1%. Regarding the children’s dental visit pattern, parents whose children visited the dentist within < 6 months had a higher prevalence of mild DA (59.6%). Conversely, those whose children who visited the dentist > 12 months ago had a higher prevalence of severe PDA (38.4%).

**Table 2:** The association of participant’s characteristics with revised version of Dental anxiety scale (DAS-R)

	Revised version of Dental Anxiety Scale (DAS-*R*)				
Variables	Mild *n* (%)	Moderate *n* (%)	High *n* (%)	Severe *n* (%)	*P*
Relationship to Child					
Mother	51 (35.4)	44 (30.6)	15 (10.4)	34 (23.6)	1.000
Father	75 (35.5)	60 (28.4)	24 (11.4)	52 (24.6)	
Caregiver/guardian	27 (36.0)	22 (29.3)	8 (10.7)	18 (24.0)	
Age of parent (years)					
20–25	18 (43.9)	10 (24.4)	8 (19.5)	5 (12.2)	< 0.001
25–30	56 (31.5)	71 (39.9)	10 (5.6)	41 (23.0)	
> 30	79 (37.4)	45 (21.3)	29 (13.7)	58 (2.5)	
Family income					
Low	0 (-)	0 (-)	3 (33.3)	6 (66.7)	0.007
Medium	70 (28.7)	96 (39.3)	22 (9.0)	56 (23.0)	
High	83 (46.9)	30 (16.9)	22 (12.4)	42 (23.7)	
Maternal education					
Primary	2 (28.6)	3 (42.9)	2 (28.6)	0 (0)	0.020
High school	29 (26.1)	42 (37.8)	15 (13.5)	25 (22.5)	
Graduate	122 (39.1)	81 (26.0)	30 (9.6)	79 (25.3)	
Presence of ≥ 1 untreated decayed teeth					
Yes	108 (30.2)	115 (32.1)	36 (10.1)	99 (27.7)	< 0.001
No	45 (62.5)	11 (15.3)	11 (15.3)	5 (6.9)	
Last child dental appointment					
< 6 months	31 (59.6)	4 (7.7)	6 (11.5)	11 (21.2)	< 0.001
6 months – 1- year	102 (44.9)	76 (33.5)	14 (6.2)	35 (15.4)	
> 1- year	20 (13.2)	46 (30.5)	27 (17.9)	58 (38.4)	

*Significantly different (*P* < 0.05): Chi-square/Fisher exact test

Table 3 provides an overview of the participants’ characteristics based on the mean OHL scores measured by REALD-30. The results revealed a significant difference (*P* < 0.05) in the OHL mean scores across all independent variables. Mothers and parents above 30 years old showed the highest mean OHL score (12.91 and 13.12, respectively), followed by those in the 25–30 (11.17) and 20–25 (7.10) age groups. Regarding income groups, high-income parents exhibited the highest mean OHL score (27.00), followed by medium-income (12.55) and low-income groups (10.59). In terms of maternal education, those with primary education showed a significantly lower mean OHL score (9.85) than those with high school (12.35) and graduate-level (14.43) education. Parents of children with no decayed teeth had a significantly higher mean OHL score (15.79) than those whose children had ≥ 1 untreated dental decay (10.92). Furthermore, children who visited the dentist within < 6 months had a significantly higher mean OHL score (17.5).

**Table 3:** Mean difference of oral health literacy (OHL) measured by Rapid Estimate of Adult Literacy in Dentistry (REALD-30)

Variables	Mean (SD)	F/t (DF)	*P*
Relationship to Child			
Mother	12.91 (5.94)*	4.77 (2)	0.009
Father	11.33 (5.99) *		
Caregiver/guardian	10.64 (5.03)		
Age of parent (years)			
20–25	7.10 (2.48) *	21.33 (2)	< 0.001
> 25–30	11.17 (4.56) *		
> 30	13.12 (6.73) *		
Family income			
Low	10.59 (5.18) *	44.29 (2)	< 0.001
Medium	12.55 (5.68) *		
High	27.00 (2.60) *		
Maternal education			
Primary	9.85 (5.53)*	8.50 (2)	< 0.001
High school	12.35 (5.89) *		
Graduate	14.43 (2.70) *		
Presence of ≥ 1 untreated decayed teeth			
Yes	10.92 (5.66)	-7.13 (107.54)	< 0.001
No	15.79 (5.21)		
Last child dental appointment			
< 6 months	17.50 (4.42) *	47.81 (2)	< 0.001
6 months – 1- year	12.09 (5.33) *		
> 1- year	9.23 (5.58) *		

*Significantly different (*P* < 0.05) after Bonferroni correction

Table 4 presents the logistic regression analysis results, in which four independent variables (parent’s age, family income, presence of ≥ 1 untreated decayed tooth and most recent child dental appointment) were retained in the final model, indicating their significance as predictors of PDA. Parents aged between 20 and 25 years were found to be 84% less likely to experience DA compared with those above 30 years (AOR = 0.16; *P* < 0.001). Parents with medium family income were approximately 2.6 times more likely to have PDA than those in the high-income group (AOR = 2.56; *P* < 0.001). Moreover, the presence of ≥ 1 untreated decayed tooth in children increased the likelihood of PDA amongst parents by approximately 2.5 times compared with those whose children had no caries (AOR = 2.45; *P* = 0.003). Parents of children who visited the dentist within < 6 months were 93% less likely to have DA than those whose children visited the dentist > 12 months ago (AOR = 0.07; *P* < 0.001). Similarly, parental DA in children who had dental appointments between 6 and 12 months ago was 86% lower than those whose children had dental appointments > 12 months ago (AOR = 0.14; *P* < 0.001).

**Table 4:** Risk indicators associated with parental dental anxiety

Variables	COR (95% CI)	*P*	AOR (95% CI)	*P*
Relationship to Child				
Mother	1.03 (0.57, 1.84)	0.932		
Father	1.02 (0.59, 1.77)	0.944		
Caregiver/guardian	1			
Age of parent (years)				
20–25	0.77 (0.39, 1.51)	0.437	0.16 (0.07, 0.4)	< 0.001
> 25–30	1.30 (0.86, 2.00)	0.218	0.98 (0.57, 1.68)	0.944
> 30	1		1	
Family income				
Low	-		-	
Medium	2.19 (1.46, 3.29)	< 0.001	2.56 (1.52, 4.33)	< 0.001
High	1		1	
Maternal education				
Primary	1.61 (0.31, 8.41)	0.575		
High school	1.82 (1.12, 2.94)	0.015		
Graduate	1			
Presence of ≥ 1 untreated decayed teeth				
Yes	3.86 (2.28, 6.54)	< 0.001	2.45 (1.37, 4.37)	0.003
No	1		1	
Last child dental appointment				
< 6 months	0.10 (0.05, 0.21)	< 0.001	0.07 (0.03, 0.18)	< 0.001
6 months – 1- year	0.19 (0.11, 0.32)	< 0.001	0.14 (0.07, 0.26)	< 0.001
> 1- year	1		1	

Table 5 presents the results of the logistic regression analysis of OHL using four independent variables: parent’s age, family income, presence of ≥ 1 untreated decayed tooth and most recent child dental appointment as predictors. Parents aged 20–25 years were 81% less likely to have OHL than those above 30 years (AOR = 0.19; *P* = 0.038). Similarly, parents with medium family income had about 52% lower likelihood of OHL than those in the high-income group (AOR = 0.48; *P* = 0.013). The presence of ≥ 1 untreated decayed tooth in children reduced the likelihood of OHL by 90% (AOR = 0.10; *P* < 0.001). Parents of children who visited the dentist within < 6 months were 34 times more likely to have OHL than those whose children visited the dentist > 12 months ago (AOR = 34.94; *P* < 0.001). Conversely, parents with children who visited the dentist within 6 months to 1 year were 2.98 times more likely to have OHL than those whose children visited the dentist > 12 months ago (AOR = 2.98; *P* = 0.001).

**Table 5:** Risk indicators associated with parental oral health literacy

Variables	COR (95% CI)	*P*	AOR (95% CI)	*P*
Relationship to Child				
Mother	1.94 (1.06, 3.55)	0.031	-	-
Father	1.10 (0.61, 1.96)	0.762	-	-
Caregiver/guardian	1			
Age of parent (years)				
20–25	0.06 (0.02, 0.27)	< 0.001	0.19 (0.04, 0.91)	0.038
> 25–30	0.49 (0.32, 0.74)	0.001	0.61 (0.34, 1.11)	0.104
> 30	1		1	
Family income				
Low	-	-	-	-
Medium	0.35 (0.23, 0.54)	< 0.001	0.48 (0.27, 0.86)	0.013
High	1		1	
Maternal education				
Primary	1.15 (0.25, 5.24)	0.854	-	-
High school	0.34 (0.20, 0.58)	< 0.001	-	-
Graduate	1			
Presence of ≥ 1 untreated decayed teeth				
Yes	0.09 (0.05, 0.16)	< 0.001	0.10 (0.05, 0.22)	< 0.001
No	1		1	
Last child dental appointment				
< 6 months	58.29 (22.35, 151.98)	< 0.001	34.94 (12.50, 97.72)	< 0.001
6 months – 1- year	5.53 (3.04, 10.05)	< 0.001	2.98 (1.55, 5.72)	0.001
> 1- year	1		1	

## Discussion

This study determined the parents’/caregivers’ DA and OHL associated with the sociobehavioural characteristics of family, self-reported child oral health (presence of ≥ 1 untreated decayed teeth) and dental visit patterns amongst children residing in Al Jouf Province, KSA. Such data are essential because parents in KSA report a higher PDA than many other countries [7, 9, 22]. High levels of PDA contribute to unfavourable patterns of dental service utilisation by affected families and constitute a significant burden on society. Moreover, the prevalence of dental caries was very high amongst Saudi school children compared with children from other developed and developing nations in the Mediterranean region [2]. The current study indicated that parents under the age group of < 25 years had a lesser prevalence of severe DA than other parental age groups. This finding suggests that parents’ age may play a role in their anxiety levels. In particular, younger parents may have less experience and knowledge about dental care, which can lead to lower anxiety levels. Moreover, parents older than 30 years had a significantly higher mean OHL score. This finding indicates that age may play a significant role in parental OHL levels, with older parents having more knowledge, awareness about oral health and ability to influence parental attitudes towards their children’s oral health.

In the present study, parents from low-income families had a high prevalence of severe DA. However, the AOR cannot confirm this finding due to the small number of participants in this income category. Nevertheless, the study’s result was evident in families with a medium income. This can be explained by the fact that despite the availability of free dental services provided by the Ministry of Health in KSA for the Saudi population, parents with low-to-medium income may still face financial barriers that limit their access to dental care, leading to higher anxiety levels. Similar findings have been reported in other studies, where SES is considered a significant indicator for regular child dental visits amongst preschool children in countries, such as Brazil and the UAE. Such observations highlight the impact of economic factors on oral-health behaviours and anxiety levels in parents [23–25].

Maternal education has been shown in the present study to have a significant association with DA, which can positively influence the oral health of the child. Education plays a crucial role in shaping parents’ knowledge and attitudes towards their children’s oral health [16]. Studies have indicated that mothers with lower levels of education tend to make less frequent dental visits for their children [26]. Conversely, mothers with higher education levels are more likely to possess better knowledge and awareness about oral health and dental care, leading to lower anxiety levels.

Maternal DA also serves as an indicator of child oral health and utilisation of dental services as well as a predictor of children’s dental behaviour [26]. A study in an eastern province of the KSA demonstrated a higher prevalence of DA amongst Saudi mothers, as well as found a significant relationship between maternal DA and untreated tooth decay in children [5]. The risk indicators of DA vary for each individual and are influenced by social backgrounds, personal experiences, feelings, expectations and aspirations [27]. Therefore, raising oral-health awareness amongst anxious parents is crucial in increasing dental care utilisation, promoting regular dental visits and reducing the burden of dental caries in children. By addressing maternal DA, oral healthcare professionals can work towards improving the overall oral health outcomes for parents and children.

In the current study, a high level of OHL was associated with maternal education at the graduate level. OHL has been found to be significantly associated with maternal social status and the dental health of the child [28]. Parents with lower levels of OHL exhibited behaviours that were less conducive to their child’s health and were less capable of advocating for their child’s oral-health needs [11, 15]. A study involving caregivers from Brazil has also reported common occurrences of poor OHL, low education and poor perceptions of children’s oral health [11]. Therefore, such a finding highlights the importance of measuring OHL and designing strategies to improve it given that it can positively influence the patient–professional relationship in oral healthcare. Low parental health literacy and low SES are associated with negative impacts on child health. Conversely, improved parental health literacy and higher SES may contribute to better outcomes in areas, such as nutrition, exercise and dental health [25]. In the present study, data indicated that low-income families had significantly lower OHL scores. This finding suggests that SES can influence OHL levels in low-income families because they may face greater barriers in accessing oral health-related information and resources. A study in Germany also reported that high parental health literacy is significantly associated with high SES and older parental age. This result emphasises the role of socioeconomic factors in shaping OHL amongst parents [25].

In our work, a significant proportion of parents (83.3%) reported that their children had ≥ 1 untreated decayed tooth. Furthermore, parents whose children had untreated dental decay demonstrated moderate and severe PDA and lower levels of OHL compared with parents of children with no cavities. The presence of a high percentage of untreated dental caries in children was concerning because it can cause pain and negatively impact a child’s quality of life. These findings suggest that OHL can play a crucial role in promoting good oral health status amongst children. However, the existing literature is inconclusive regarding the relationship between OHL and dental treatment outcomes, oral health behaviours and caregivers’ perceptions of oral health associated with OHL. Thus, to enhance our understanding and improve oral-health outcomes in children, further research is needed to explore and elucidate the association between OHL and these important aspects of dental care [29, 30].

The present study revealed a significant association between the pattern of child dental visits and PDA levels. Parents of children who visited the dentist within > 6 months had a significantly higher mean OHL score. DA is an important variable linked to the utilisation of dental services in children [29]. Regular dental visits can reportedly reduce PDA levels and promote better oral health outcomes for children [31]. Similar patterns have been observed in Malaysia, where demographic and socioeconomic factors strongly influence oral healthcare utilisation [32]. In the KSA, oral health service utilisation may be influenced by various factors, including the cost of treatment, accessibility to dental services, waiting periods for dental appointments, level of parental education and the presence of severe symptoms, such as pain [33]. Given that these factors collectively contribute to the use of dental services, understanding these variables can aid in developing strategies to improve access to oral healthcare services and reduce DA amongst parents and caregivers, thus leading to better oral-health outcomes for children.

This study has certain limitations that must be considered. First, being a cross-sectional study, we cannot establish a temporal relationship between the outcome and exposure variables simultaneously. Second, the study participants were high-risk children attending a university dental centre, which may lead to skewed results. The fact that parents included in this study were dental clinic visitors can cause selection bias in the evaluation of dental anxiety. We emphasise that our sample may not fully represent the general population. Moreover, the use of self-reported questionnaires may have led to some degrees of distortion and bias, such as recall inaccuracies and biases influenced by social desirability. Despite these limitations, our work has important clinical implications for paediatric dental practice. Dentists are recommended to assess PDA levels, OHL and sociobehavioural characteristics of parents during a child’s first dental visit using appropriate scales and semistructured interviews.

Furthermore, oral healthcare providers should inquire about parents’ previous dental experiences and perceptions of dental treatments, encourage them to ask questions and address any anxiety they may have regarding these visits. Our findings can serve as a foundation for further prospective, longitudinal and population-based studies to identify significant risk factors associated with parental anxiety in the Saudi population. Future research can help elucidate the complex factors influencing DA and pave the way for more targeted interventions and improved oral health outcomes in Saudi children.

In addition, this study identified significant predictors of PDA, including parental age, family income, presence of ≥ 1 untreated decayed tooth in children and the most recent child dental visit. Parental anxiety was observed to contribute to an unfavourable pattern of dental service utilisation amongst affected families. Therefore, parents and caregivers often require assistance in improving their awareness and OHL, especially amongst those with low incomes and lower levels of education. Promoting a favourable pattern of dental care service utilisation at the individual, family and community levels is essential. In addition, understanding the risk indicators of PDA and OHL can aid oral healthcare professionals in recognising challenges and effectively engaging with parents to build trust and promote early and regular child dental visits, thereby improving their children’s oral health. Finally, policymakers should develop and implement effective interventions to reduce PDA and promote OHL, as well as enhance sociobehavioural characteristics to promote optimal oral health outcomes in children. By focusing on these factors and providing appropriate support and interventions, oral healthcare professionals can work towards enhancing the overall oral health of children and creating a positive impact on their dental care experiences.

## Conclusion

Within the limitations of this study, factors including the age of parents, family income, presence of ≥ 1 untreated decayed tooth in children and most recent child dental visit were identified as the predictors of PDA and OHL. Therefore, during a child’s first dental visit, paediatric dentists should always assess the PDA, OHL and sociobehavioural characteristics of the family by using appropriate scales and semistructured interviews.

## Data Availability

The datasets used and/or analysed during the current study available from the corresponding author on reasonable request.
